# The Effect of the Non-compressed Oxygen Therapy and Hyperbaric Oxygenation in Combination With Standardized Drug Therapy on the Blood Acid-Base State Biomarkers in Alcohol Withdrawal Syndrome, an Experimental Study

**DOI:** 10.3389/fpsyt.2022.819154

**Published:** 2022-04-18

**Authors:** Dzmitry Kapytau, Andrei Kapytau, Inessa Khrushch, Ludmila Kudin, Napoleon Waszkiewicz

**Affiliations:** ^1^Department of Psychiatry and Medical Psychology, Belarusian State Medical University, Minsk, Belarus; ^2^Department of Clinical and Diagnostic Laboratory, Belarusian State Medical University, Minsk, Belarus; ^3^Department of Psychiatry, Medical University of Białystok, Białystok, Poland

**Keywords:** alcohol withdrawal syndrome, acid-base, markers, hyperbaric oxygenation, treatment, non-compressed oxygen therapy

## Abstract

In alcohol withdrawal syndrome (AWS), pathophysiological mechanisms cover acid-base disturbances that affect the clinical picture of this state. An earlier study found that oxygen therapy methods in combination with pharmacotherapy improved the cognitive state in persons suffering from AWS. As impairments in the acid-base state influence the general health, timely and effective correction of these acid-base disturbances could result in a potential improvement in the treatment of the alcohol withdrawal symptoms. Therefore, the aim of this study was to evaluate the effectiveness of non-compressed oxygen therapy (NOT) and hyperbaric oxygenation (HBO) in combination with standard drug therapy (SDT), based on the dynamics of the acid-base state (ABS) in blood during AWS. HBO is the use of oxygen under pressure, whereas NOT uses oxygen without pressure. A comparative assessment of the acid-base state biomarkers was made in 160 patients with a moderate alcohol withdrawal state (3 groups), namely, in patients who underwent SDT only (control group/CG; *n* = 42) and two comparison groups who underwent SDT in combination with NOT (SG1 group; *n* = 56) and HBO (SG2 group; *n* = 62). The use of both oxygen therapy methods (i.e., NOT and HBO) in combination with SDT corrected the ABS in a shorter time and more effectively, which was due to the better restoration of the carbonate buffer system. Although we did not find proof that novel oxygen-related therapeutic procedures such as NOT and HBO in combination with SDT improved the alcohol withdrawal symptoms, it helped with the faster restoration of the acid-base state.

## Introduction

Alcohol withdrawal syndrome (AWS) is a result of the several pathophysiological mechanisms. After a sharp cessation of alcohol consumption, the inhibitory effect of ethanol on the central nervous system ends, which leads to an increase in the excitatory effect of glutamate and changes in neurotransmitter systems (1–4). In AWS, there are disturbances in energy homeostasis and a decrease in brain tissue reserves, which seems to be partly due to deregulated brain metabolism (3, 5).

Active transmembrane electrolyte transport is one of the most important components of the energy-dependent process. Inorganic electrolytes provide up to 96% of the total osmotic pressure of blood (5, 6). The electrochemical balance of ions between the outer and inner layer of the membrane creates its potential. Energy potential of cells is based on the universal process of glucose breakdown, but with a lack of oxygen in cells of the body, accumulation of lactate begins, which is a toxic substance. As a result, lactic acidosis develops, causing the dysfunction of many cells, primarily nerve and muscle cells (6–9). It was found that, with AWS, the body's need for oxygen increases, which decreases with the decline in the severity of clinical symptoms (5). In AWS, oxygen deficiency is predominantly of the tissue type, as evidenced by the low arteriovenous oxygen difference. Presumably, this may be due to the effect of alcohol breakdown products on the dehydrogenase system, which subsequently causes cerebral tissue hypoxia with subsequent disturbances in the mental sphere (6).

Patients with alcohol dependence (AD) syndrome have lower serum concentrations of potassium, magnesium, bicarbonate, calcium, and phosphate as well as a lower arterial pH. The severity of AWS correlates with the dynamics of the ABS markers, circulating blood volume, and water-electrolyte balance. Also with AWS, disturbances in homeostasis may occur with acidosis or alkalosis. The causes of the ABS disturbances can be respiratory, metabolic, or a combination of both. Acidosis is a deviation of ABS, characterized by an excessive content of acid anions in the blood and tissues with a decrease in pH (9–13).

Alcohol withdrawal syndrome of any severity requires mandatory treatment for the prevention of severe complications as epileptic seizures, Wernicke encephalopathy or dementia, and the treatment of already developed disorders as malnutrition or electrolyte and acid-base imbalance (13). For a successful treatment of AWS, in addition to relieving psychopathological, somato-neurological, and autonomic symptoms, it is desirable to replenish the fluid deficit, to correct water-electrolyte disorders and acid-base disorders (13). The use of standard treatment for AWS, which contains predominantly psychotropic drugs, does not always take into account changes in the ABS, especially in moderate AWS, when medical care is provided mainly outside of intensive care units.

Hyperbaric oxygen (HBO) therapy involves the use of oxygen under pressure to facilitate tissue healing and has been used routinely for over 40 years in the medicine. Currently, HBO is a well-established therapy for several pathological states including air or gas embolism, carbon monoxide poisoning, decompression sickness, and wounds/soft tissue infections (14). Tissue oxygen disturbances and cell dysfunctions/degenerations are also considered as the etiology of neuropsychiatric disorders, e.g., post-traumatic stress disorder, major depression, traumatic brain injury, or autism spectrum disorder, as a target of HBO (14, 15).

As disturbances in ABS can influence overall health (16), we hypothesized that methods of oxygen therapy can also be one of additional methods that positively affect ABS disturbances during the AWS, which potentially can improve AWS. The effect of oxygen therapy on ABS in persons suffering from AWS has not been studied so far. Biochemical parameters such as pH, carbon dioxide tension, bicarbonate, Cl–, lactate, and blood base excess (BE) are called acid-base markers (16). Therefore, the aim of this study was to evaluate the effectiveness of HBO and non-compressed oxygen therapy (NOT) methods in combination with standard drug therapy (SDT) on the dynamics of biological markers of ABS during AWS. The NOT group was used to compare the effect of hyperbaric with uncompressed oxygenation.

## Materials and Methods

### Participants

A total of 160 male patients with moderate AWS were examined. The study group was recruited from people successively admitted to the hospital for the treatment of the AWS. All of the patients suffered from AD and were inpatients receiving treatment at the Republican Scientific and Practical Center for Mental Health in Belarus: 62 people underwent HBO (SG2) and 56 NOT (SG1) together with SDT and 42 underwent only SDT in accordance with the protocols of medical help (CG). Biochemical analyses were carried out on the 1st, 3rd, and 7th day of the treatment.

The most recent alcohol consumption of the subjects took place the day before admission to inpatient treatment. Clinical examination of the somatic status was carried out according to the generally accepted scheme. The exclusion criteria were the presence of chronic diseases that might influence ABS biomarkers or contraindications in the HBO: non-alcoholic liver disease, acute and chronic pancreatitis, chronic renal failure, viral hepatitis, metabolic diseases (diabetes), obstruction of the Eustachian tubes, increased body temperature, epilepsy, claustrophobia, oncological diseases, acute inflammatory processes, severe cardiovascular disease, bleeding and trauma, and addiction to psychoactive substances other than ethanol.

For the SDT treatment of the AWS, parenterally and orally, diazepam was used, in a dose not exceeding 40 mg/day. In addition, intravenous natrium chloratum injections, multielectrolyte fluids, and glucose were used. In the HBO group, the patients received an HBO session one time a day for 7 days. The pressure in the HBO chamber reached 0.2 MPa (2.0 ATA) and lasted 30 min. At the end of treatment, the pressure gradually decreased over a period of 10–15 min. The patients in the NOT group were, similarly to HBO, put into an HBO chamber one time a day for 7 days, but the NOT patients received oxygen without pressure for the same time period.

### Ethical Issues

The study was approved by the local Bioethical Committee of Belarusian State Medical University (N 8/124.15.08.2017) and conducted in accordance with the Helsinki Declaration. Informed written consent was obtained from all the subjects after explaining the nature, purpose, and potential risks of the study.

### Procedures

#### Data and Sample Collection

To assess the severity of AWS symptoms, Clinical Institute for Withdrawal Assessment-for Alcohol (CIWA-A) scale was used (17). In patients of all the study groups at the time of the first medical intervention, the CIWA scores were in the range of 16–20 points (no statistical differences), which corresponded to moderate AWS and was one of the significant criteria for inclusion in the study.

Clinical verification and diagnosis of AD were carried out by qualified specialists in accordance with the diagnostic (research) criteria of International Classification of Diseases (ICD)-10 (18) (presence of at least 3 out of 6 criteria for AD, observed within 1 month or periodically repeated within 12 months, objectified by two independent sources) and indicators >20 points on the Alcohol Use Disorders Identification Test (AUDIT) (19).

The study of anamnestic data, including the previous features of the course of disease, assessment of the quality, and effectiveness of the clinical dynamics of AWS, was carried out by using the Belarusian index of severity of addiction for clinical use and training (“B-ITA,” version 2.3-3.01.2001) and “scales of dynamics of psychopathological disorders in AWS, post-withdrawal state, remission” (6, 20). The dynamics of the clinical symptoms of AWS were assessed every day from the first day of admission in the hospital.

#### Analytical Methods

The biochemical study included an assessment of blood ABS with the analysis of its most relevant components: pH, partial pressure of carbon dioxide oxygen in the blood (i.e., pCO_2_ and pO_2_), saturation (i.e., SO_2_), levels of hemoglobin, glucose, Ca^++^, Na^+^, actual bicarbonate (HCO^3−^), extracellular fluid base excess/base excess (BEecf), blood base excess/actual base excess (BEb), standard bicarbonate (SB), total blood oxygen concentration (O_2_Ct), oxygenation (O_2_Cap), and alveolar-arterial gradient (D(A-a)O_2_). The abovementioned parameters were determined by using the routine laboratory methods on the Osmotech OPTI Blood Gas Analyzer. ABS biomarkers were evaluated on the 1st, 3rd, and 7th day of the treatment.

### Statistical Analysis

Statistical processing of the research results was carried out by using the statistical software package STATISTICA 10.0 (SN: BXXR207F383502FA-D). To assess the normality of distribution, the Kolmogorov-Smirnov test was used. Taking into account the normality of the distribution, the methods of parametric statistics were used. A comparison of the mean values between groups was carried out by using one-way analysis of variance (ANOVA with *post-hoc* tests) and within the group (between 1st, 2nd, and 7th day-dependent variables) by using the *t*-test. A comparison of the groups by a qualitative binary feature was carried out by using the χ^2^ criterion, and the odds ratio was calculated. A mixed design ANOVA (3 time-points response × 3 groups) was used to test the differences in acid-base disturbances over the course of the 1st, 3rd, and 7th day of treatment between the SG1 (1), SG2 (2), and CG (3) groups. The differences between groups were considered significant at *p* < 0.05.

## Results

The average age of the patients in study groups did not differ significantly between them and was, respectively: 38.3 ± 1.3 years in SG1; 40.2 ± 1.3 years in SG2; and 38.8 ± 1.3 years in CG. Participants were from rural (39.7%) and urban (60.3%) areas. The educational level was secondary (30.6%), secondary specialized (56.5%), and higher (12.9%). There were no statistically significant differences between the groups in the time of nicotine dependence and number of smoked cigarettes per day (from 94 to 97% were smokers). The time of AD in the groups was: in CG, 17.6 ± 2.2 years; in SG2, 20.9 ± 2.6 years; and in SG1, 16.5 ± 2.1 years (mean ± SD). The average duration of the most recent chronic alcohol drinking period was: in the control group, 14.6 ± 1.7 days; in SG2, 14.6 ± 1.7 days; and in SG1, 13.5 ± 2.3 days, with no statistical differences.

The analysis of the levels of blood ABS indicators presented in Table 1 showed that most of them (except of glucose and Na^+^) underwent significant changes during the treatment. Some of these indicators for most of the subjects were within the range of standard values. The data were analyzed in two ways, namely, within-group and between-group differences.

**Table 1:** The dynamic changes of blood acid-base parameters.

**Index**	**Groups**			***P***
	**SG1 (1)**	**SG2 (2)**	**CG (3)**	
pH after 1 day of therapy	7.505 ± 0.007	7.522 ± 0.007	7.536 ± 0.005	P_1, 2−3_ <0.05
pH after 3 days of therapy	7.499 ± 0.006	7.503 ± 0.005	7.513 ± 0.004	P_1, 2−3_ <0.05
pH after 7 days of therapy	7.484 ± 0.005	7.481 ± 0.006	7.507 ± 0.003	P_1, 2−3_ <0.05
pCO_2_ after 1 day of therapy (mmHg)	38.09 ± 0.72	39.23 ± 0.90	35.44 ± 0.83	P_1, 2−3_ <0.05
pCO_2_ after 3 days of therapy (mmHg)	38.97 ± 0.79	39.83 ± 1.03	35.90 ± 0.91	P_1, 2−3_ <0.05
pCO_2_ after 7 days of therapy (mmHg)	40.22 ± 0.88	38.92 ± 0.85	36.07 ± 0.95	P_1, 2−3_ <0.05
pO_2_ after 1 day of therapy (mmHg)	74.61 ± 1.68	80.63 ± 1.53	73.23 ± 2.02	P_2−1, 3_ <0.05
pO_2_ after 3 days of therapy (mmHg)	78.38 ± 1.38	82.62 ± 2.00	71.59 ± 1.73	P_1, 2−3_ <0.05
pO_2_ after 7 days of therapy (mmHg)	78.82 ± 1.39	82.42 ± 1.73	70.36 ± 2.67	P_1, 2−3_ <0.05
SO_2_ after 1 day of therapy (%)	96.76 ± 0.19	97.37 ± 0.26	94.17 ± 1.37	P_1, 2−3_ <0.05
SO_2_ after 3 days of therapy (%)	96.45 ± 0.31	97.23 ± 0.34	95.07 ± 0.39	P_1, 2−3_ <0.05
SO_2_ after 7 days of therapy (%)	96.56 ± 0.31	97.46 ± 0.30	94.92 ± 0.45	P_1, 2−3_ <0.05
Hemoglobin after 1 day of therapy, g/l	148.4 ± 1.73	156.2 ± 1.75	155.4 ± 1.99	P_1−2, 3_ <0.05
Hemoglobin after 3 days of therapy, g/l	143.9 ± 1.91	154.7 ± 2.06	150.6 ± 1.98	P_1−2, 3_ <0.05
Hemoglobin after 7 days of therapy, g/l	143.3 ± 1.73	152.6 ± 1.93	148.2 ± 1.96	P_1−2, 3_ <0.05
Ca^++^ after 1 day of therapy, mmol/l	1.00 ± 0.02	0.98 ± 0.02	0.89 ± 0.02	P_1, 2−3_ <0.05
Ca^++^ after 3 days of therapy, mmol/l	1.01 ± 0.01	0.99 ± 0.02	0.90 ± 0.02	P_1, 2−3_ <0.05
Ca^++^ after 7 days of therapy, mmol/l	1.00 ± 0.01	1.00 ± 0.02	0.93 ± 0.01	P_1, 2−3_ <0.05

*pH, Potential of hydrogen; pCO_2_, partial pressure of carbon dioxide; pO_2_, partial pressure of oxygen; SO_2_, oxygen saturation; SDT, level of hemoglobin, glucose, and Ca^2+^ and Na^+^ in the blood of persons suffering from an alcohol withdrawal state (AWS) that had standardized drug therapy; NOT, non-compressed oxygen therapy; HBO, hyperbaric oxygenation; CG, patients who underwent SDT only (control group); SG1, patients who underwent SDT in combination with NOT; SG2, patients who underwent SDT in combination with HBO; P1, 2, −3 < 0.05 means statistically significant difference between the SG1 (1) and CG (3) groups, statistical difference between SG2 (2) and CG(3) groups, and no differences between the SG1 (1) and SG2 (2) groups; P2–1, 3 < 0.05 means statistically significant difference between the SG1 (1) and SG2 (2) groups, statistical difference between CG(3) and SG2 (2) groups, and no differences between the SG1 (1) and CG (3) groups; P1–2, 3 < 0.05 means statistically significant difference between the SG1 (1) and SG2 (2) groups, statistical difference between SG1 (1) and CG(3) groups, and no differences between the SG2 (2) and CG (3) groups; p < 0.05 at the bottom of each group means statistically significant difference in parameter during the 7-day treatment period. There were found intragroup differences only in the pH values in SG1, SG2, and CG groups*.

Although 7 days of therapy in groups with oxygen methods (i.e., NOT and HBO) significantly changed the pH values, its levels did not differ significantly for more than +0.1 from the normal range.

In addition to the main ABS indicators (Table 1), Table 2 presents indicators that show mainly compensatory resources and the capabilities of the body in certain biochemical disorders. The average levels of bicarbonate (HCO^3−^) in all the groups are higher than the reference ranges (7.35–7.45). Although we found a significant difference during the 7-day period (within-group) of AWS only in HCO_3_ in the CG group (Table 2), we found statistical differences between groups in all the parameters at all the time points of the study.

**Table 2:** The dynamic changes of the acid-base state parameters.

**Index**	**Groups**			***P***
	**GS1 (1)**	**GS2 (2)**	**CG (3)**	
HCO_3_- after 1 day of therapy, mmol/l	32.08 ± 0.73	32.28 ± 0. 71	28.23 ± 0.68	P_1, 2−3_ <0.05
HCO_3_- after 3 days of therapy, mmol/l	31.55 ± 0.52	31.16 ± 0.72	27.63 ± 0.76	P_1, 2−3_ <0.05
HCO_3_- after 7 days of therapy, mmol/l	31.67 ± 0.59	31.57 ± 0. 71	27.17 ± 0.71	P_1, 2−3_ <0.05
BEecf after 1 day of therapy, mmol/l	9.87 ± 0.59	9.08 ± 0.76	4.92 ± 0.73	P_1, 2−3_ <0.05
BEecf after 3 days of therapy, mmol/l	9.87 ± 0.59	9.08 ± 0.76	4.92 ± 0.73	P_1, 2−3_ <0.05
BEecf after 7 days of therapy, mmol/l	8.55 ± 0.52	8.19 ± 0.74	3.49 ± 0.74	P_1, 2−3_ <0.05
BEb after 1 day of therapy, mmol/l	9.86 ± 0.48	9.26 ± 0.61	5.83 ± 0.61	P_1, 2−3_ <0.05
BEb after 3 days of therapy, mmol/l	8.58 ± 0.42	7.96 ± 0.61	4.63 ± 0.66	P_1, 2−3_ <0.05
BEb after 7 days of therapy, mmol/l	8.59 ± 0.41	8.38 ± 0.58	4.32 ± 0.62	P_1, 2−3_ <0.05
SB after 1 day of therapy, mmol/l	33.69 ± 0.48	33.15 ± 0.62	29.62 ± 0.59	P_1, 2−3_ <0.05
SB after 3 days of therapy, mmol/l	31.80 ± 0.67	31.83 ± 0.59	28.53 ± 0.63	P_1, 2−3_ <0.05
SB after 7 days of therapy, mmol/l	32.37 ± 0.39	32.26 ± 0.57	28.11 ± 0.63	P_1, 2−3_ <0.05
O_2_Ct after 1 day of therapy, ml/dl	20.20 ± 0.24	21.41 ± 0.26	20.76 ± 0.30	P_2−1, 3_ <0.05
O_2_Ct after 3 days of therapy, ml/dl	19.65 ± 0.39	21.15 ± 0.31	20.76 ± 0.30	P_2−1, 3_ <0.05
O_2_Ct after 7 days of therapy, ml/dl	19.48 ± 0.25	20.93 ± 0.28	19.77 ± 0.26	P_2−1, 3_ <0.05
O_2_Cap after 1 day of therapy, ml /dl	20.63 ± 0.24	21.71 ± 0.24	21.51 ± 0.30	P_1−2, 3_ <0.05
O_2_Cap after 3 days of therapy, ml/dl	20.01 ± 0.27	21.47 ± 0.29	20.93 ± 0.27	P_1−2, 3_ <0.05
O_2_Cap after 7 days of therapy, ml/dl	19.92 ± 0.24	21.19 ± 0.26	20.56 ± 0.27	P_1−2, 3_ <0.05
D(A-a)O_2_ after 1 day of therapy (mmHg)	23.28 ± 1.44	20.35 ± 1.72	30.32 ± 1.85	P_1, 2−3_ <0.05
D(A-a)O_2_ after 3 days of therapy (mmHg)	21.62 ± 1.33	17.93 ± 1.91	30.49 ± 1.88	P_1, 2−3_ <0.05
D(A-a)O_2_ after 7 days of therapy (mmHg)	19.49 ± 1.46	18.01 ± 1.84	29.19 ± 1.94	P_1, 2−3_ <0.05

*HCO_3_, bicarbonate level; BEecf, extracellular fluid base excess/base excess; BEb, blood base excess/actual base excess; SB, standard bicarbonate; O_2_Ct, oxygen concentration; O_2_Cap, total blood oxygenation; SDT, alveolar-arterial gradient (D(A-a)O_2_) in the blood of persons suffering from alcohol withdrawal state (AWS) that had standardized drug therapy; NOT, non-compressed oxygen therapy; HBO, hyperbaric oxygenation; CG, patients who underwent SDT only (control group); SG1, patients who underwent SDT in combination with NOT; SG2, patients who underwent SDT in combination with HBO; P1, 2, −3 < 0.05 means statistically significant difference between the SG1 (1) and CG(3) groups, statistical difference between the SG2 (2) and CG(3) groups, and no differences between the SG1 (1) and SG2 (2) groups; P2–1, 3 < 0.05 means statistically significant difference between the SG1 (1) and SG2 (2) groups, statistical difference between CG(3) and SG2 (2) groups, and no differences between the SG1 (1) and CG (3) groups; P1–2, 3 < 0.05 means statistically significant difference between the SG1 (1) and SG2 (2) groups, statistical difference between the SG1 (1) and CG(3) groups, and no differences between the SG2 (2) and CG (3) groups; p < 0.05 at the bottom of each group means statistically significant difference in the parameters during the 7-day treatment period. There were found intragroup differences only in the HCO^3−^ values in the CG group*.

**Figure 1:**
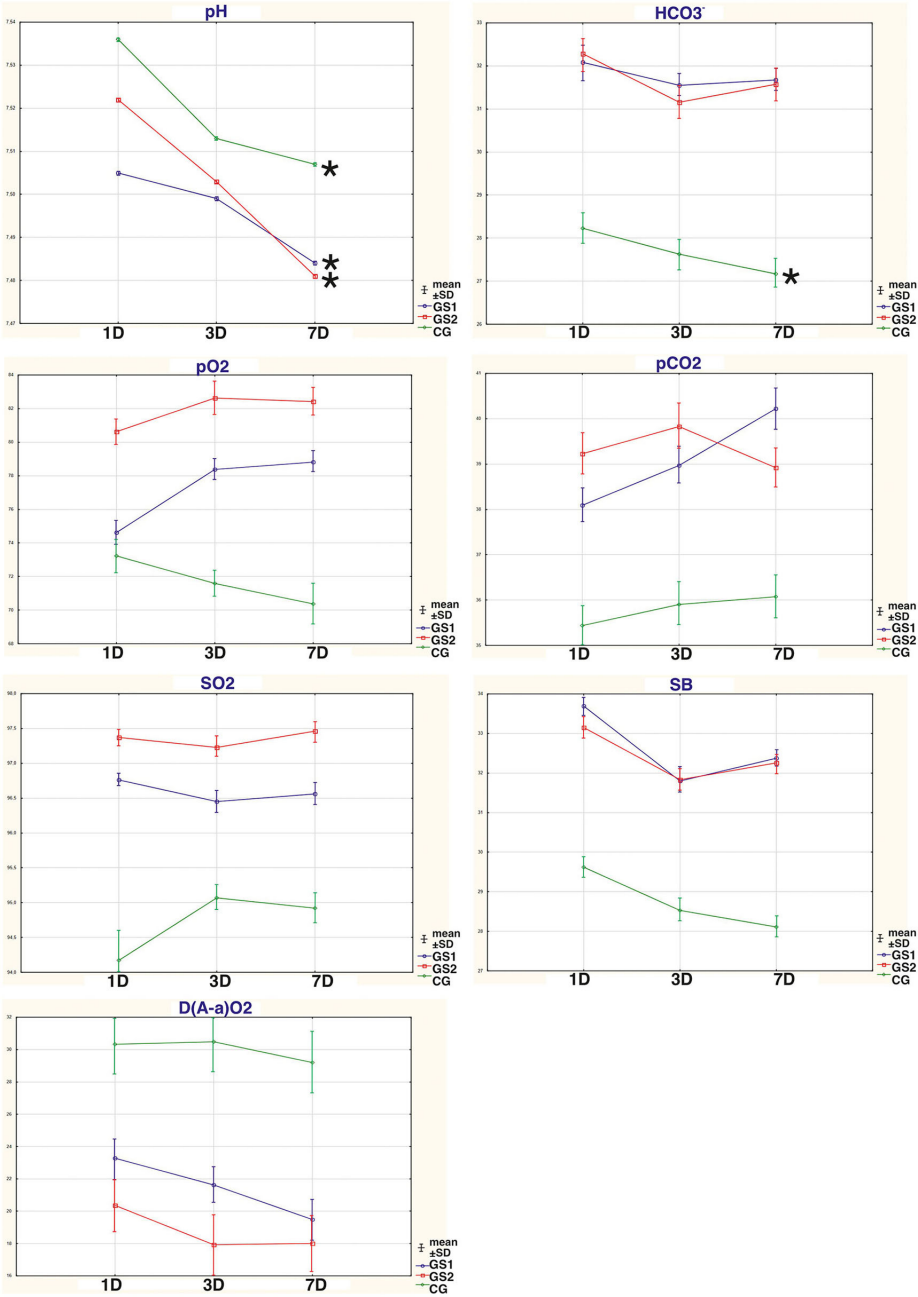
The dynamic changes in the acid-base parameters that are most important to the discussion: pH, partial pressure of carbon dioxide (pCO_2_), partial pressure of oxygen (pO_2_), oxygen saturation (SO_2_), bicarbonate level (HCO_3_), standard bicarbonate (SB), and alveolar-arterial gradient (D(A-a)O_2_), in the blood of persons suffering from AWS that had standardized drug therapy (SDT), non-compressed oxygen therapy (NOT), and hyperbaric oxygenation (HBO); CG, patients who underwent SDT only (control group); SG1, patients who underwent SDT in combination with NOT; SG2, patients who underwent SDT in combination with HBO; 1D, first day of therapy; 3D, third day of therapy; 7D, seventh day of therapy; *significant differences within the group *p* < 0.05 (*t*-test).

The results with changes in the ABS parameters that are the most important to the discussion are presented in Figure 1.

Table 3 shows that only CO_2_ values had significant effect for time, groups, and a significant time-by-group interaction but without statistical significance of changes in time in the *t*-test (Table 1). Mixed ANOVA design also showed significant interactions for time and groups in pH and O_2_Ct values with a strong tendency to significant time-by-group interaction (O_2_Ct values without significant changes in time in the *t*-test, Table 1). The effect for time in pH value had a higher effect size than for groups. It was also found a significant effect of only time for Na^+^ and a significant effect of only group for pO_2_, SO_2_, glucose, Ca^2+^, and HCO^3−^. The significant effect of time and group but without significant time-by-group interaction was found for hemoglobin, BEecf, BEb, SB, O_2_Cap, and D(a-A)O_2_.

**Table 3:** Summary of the blood acid-base state results obtained in the mixed design analysis of variance (ANOVA) with repeated measures.

**Index in 1**_**−**_ **7 days of therapy in GS1, GS2 and CG groups**		**Mixed Design ANOVA with repeated measures**			
		***F***	**df**	**η^2^**	***P***
pH effects	For time	18.0	2	0.141	**<0.0001** ^***^
	For group	0.02	2	0.125	0.0006***
	For interaction: time+ group	2.0	4	0.039	0.065
pCO_2_ effects	For time	4.0	2	0.035	0.019*
	for group	3.8	2	0.064	0.024*
	For interaction: time+ group	2.5	4	0.044	0.037*
pO_2_ effects	For time	1.1	2	0.010	0.325
	For group	9.1	2	0.142	0.0002***
	For interaction: time+ group	1.7	4	0.030	0.150
SO_2_ effects	For time	0.5	2	0.004	0.613
	For group	11.6	2	0.172	<0.0001***
	For interaction: time+ group	1.0	4	0.016	0.432
Hemoglobin effects	for time	10.7	2	0.088	<0.0001***
	For group	9.0	2	0.140	0.0002***
	For interaction: time+ group	1.4	4	0.025	0.213
Glucose effects	for time	0.9	2	0.006	0.400
	For group	3.1	2	0.043	0.047*
	For interaction: time+ group	1.6	4	0.023	0.158
Ca^++^ effects	For time	1.2	2	0.010	0.294
	for group	8.1	2	0.128	0.0004***
	For interaction: time+ group	0.6	4	0.011	0.648
Na^+^ effects	For time	4.5	2	0.039	0.011*
	For group	2.1	2	0.036	0.126
	for interaction: time+ group	0.6	4	0.009	0.693
HCO_3_- effects	For time	1.9	2	0.017	0.142
	For group	10.3	2	0.158	<0.0001***
	For interaction: time+ group	0.2	4	0.005	0.883
BEecf effects	For time	6.5	2	0.056	0.001**
	For group	14.4	2	0.207	<0.0001***
	For interaction: time+ group	0.5	4	0.009	0.720
BEb effects	For time	10.7	2	0.089	<0.0001***
	For group	15.4	2	0.219	<0.0001***
	For interaction: time+ group	0.7	4	0.012	0.576
SB effects	For time	7.7	2	0.065	0.0005***
	for group	13.7	2	0.199	<0.0001***
	For interaction: time+ group	0.7	4	0.014	0.533
O_2_Ct effects	For time	10.9	2	0.089	<0.0001***
	for group	9.6	2	0.149	0.0001***
	For interaction: time+ group	2.0	4	0.035	0.090
O_2_Cap effects	For time	10.4	2	0.085	<0.0001***
	For group	8.5	2	0.133	0.0003***
	for interaction: time+ group	1.3	4	0.023	0.267
D(A-a)O_2_ effects	For time	4.4	2	0.043	0.013*
	For group	9.4	2	0.162	0.0001***
	For interaction: time+ group	1.1	4	0.023	0.323

BEb, blood base excess/actual base excess; BEecf, extracellular fluid base excess/base excess; Ca2+, calcium; D(A-a)O_2_, alveolar-arterial gradient; HCO_3_, bicarbonate level; Na^+^, sodium; O_2_Ct, oxygen concentration; SO_2_, oxygen saturation; pCO_2_, partial pressure of carbon dioxide; pO_2_, partial pressure of oxygen; SB, standard bicarbonate; O_2_Cap, total blood oxygenation; η^2^, Eta squared effect size; F, F-value; df, degrees of freedom; significant results (*p < 0.05, **p < 0.01, and ***p < 0.001) are bolded.

Supplementary Table 1 shows the differences in the distribution of subjects (%) with the blood levels of the main indicators of ABS deviating from the reference ranges, in the GS1, GS2, and the CG groups. Statistically significant between-group differences from the reference ranges were found in pCO_2_, pO_2_, and SO_2_ but not for pH, hemoglobin, glucose, Ca^2+^, and Na^+^ values. Statistical within-group differences were located only for the number of persons (%) with indicators pO_2_ < N.

In groups where the methods of oxygen therapy were used, compared with CG, the number of subjects with low pO_2_ values decreased significantly.

The levels of ionized sodium (Na^+^) were within the reference ranges in the vast majority of subjects in all the groups, and the applied methods of treatment did not have a significant effect on this indicator.

The data in Supplementary Table 2 show statistically significant higher relative numbers of persons in SG1 and SG2, compared with the CG, with higher levels of HCO^3−^, BEecf, BEb, and SB and lower levels of D(A-a)O_2_ than the reference ranges. For parameters, such as HCO^3−^ and BEecf, this is relevant from the third day from the start of therapy, and for the rest, from the first day. Supplementary Table 2 also shows the most relevant components of the ABS, which are more actively involved in the process of the normalization of ABS.

We found no statistically significant differences between the groups or within groups in the CIWA-A scores (all the values were in the 16–20 point range) and no correlations between the CIWA-A scores and ABS markers.

## Discussion

Evidence of the positive influence of oxygen therapy methods on cognitive functions during the AWS can be found in the medical literature (1). As we hypothesized, our study showed the positive influence of oxygen therapy methods on the markers of the ABS.

Our results demonstrate that by the seventh day of therapy in groups with the oxygen therapy methods used in the complex treatment, the results of the pH levels did not differ significantly from the results exceeding the norm by +0.1. The pH index on the seventh day of therapy shows that in the SG1 and SG2 groups, there were phenomena of subcompensated alkalosis, which was not observed in CG (Figure 1, Table 1). One of the significant markers of ABS is the pH indicator (9), which represents the negative decimal logarithm of the molar concentration of hydrogen ions in cells and biological fluids. This component is one of the most important parameters for ensuring the homeostasis of the body. The bicarbonate (hydrocarbonate) buffer system is a kind of compensatory reserve resource that ensures the normalization of homeostatic equilibrium in moderate AWS of persons with AD (9). A shift in pH in the range of ±0.1 causes respiratory and circulatory disorders, ± 0.3 disturbances in hemodynamics and ventilation of the lungs, more than ± 0.4 leads to the death of the body (9, 11, 18). In arterial blood (plasma), pH ranges within 7.40 ± 0.04. All violations of ABS with the shift in the concentration of hydrogen ions are divided into acidosis and alkalosis: acidosis at pH < 7.4; alkalosis at pH > 7.4 (4, 6, 9). Therefore, subjects without oxygenation procedures (i.e., CG) might have higher circulatory disturbances at the level of decompensated alkalosis than in the SG1 and SG2 groups.

When analyzing the pH indicator, we noticed that subjects with moderate AWS had alkalosis phenomenon (Figure 1, Table 1). Given that the group mean pH values were >7.5, according to Supplementary Table 1, the vast majority of subjects had higher HCO^3−^ levels than the normal/reference ranges (N), with pCO_2_ levels close to normal or low (Table 1 and Supplementary Table 1). In these cases, it indicates the metabolic alkalosis. pH values higher than 7.5 indicate decompensated alkalosis. In all the groups, there was a statistically significant decrease in pH levels toward the normalization of this indicator, especially in groups with supplemented methods of oxygen therapy (i.e., NOT and HBO). Moreover, the advantages of any method of oxygen therapy used in the complex treatment have not been established over each other. Metabolic alkalosis is a primary disorder of the ABS, in which the level of HCO^3−^ rises, pH is higher than 7.45, and CO_2_ decreases or is normal. The primary mechanism for the shift in equilibrium in metabolic alkalosis is the loss of non-volatile acids by the body or excessive introduction of bases into the body. Clinically, such biochemical disorders can be accompanied by changes in consciousness, muscle weakness, hyporeflexia, polyuria, polydipsia, and myocardial dysfunctions (9). The use of SDT alone as well as its combination with methods of oxygen therapy did not allow for the normalization of HCO^3−^ levels, but some tendencies in the process of normalization occurred. Normalization would probably require a longer treatment period of our study. The use of oxygen therapy methods in complex treatment also reduced the number of persons with decompensated alkalosis with their transition to the stage of compensated alkalosis, respectively, in SG1 from 83 to 52.9%, in SG2 from 65.4 to 53.2% (*p* < 0.05), which was not observed in the control group.

Base excess is the metabolic component of ABS dysfunction. A positive value indicates metabolic alkalosis, a negative value indicates metabolic acidosis. The indicator of uncompensated metabolic alkalosis covers an increase in pH above 7.45 and an increase in the following values: BEecf, BEb, and SB. BE is defined as the amount of strong acid that must be added to each liter of fully oxidized blood to return the pH to 7.40 under normal conditions (9–12). The results presented in Table 2 and Supplementary Table 2 indicate that the average excess of bases in extracellular fluid, blood, and SB exceeds the reference ranges in all the groups, which also confirmed the presence of metabolic alkalosis in all the subjects from the first to the seventh day of AWS therapy. The applied methods of treatment in all the groups did not significantly affect the excess of bases for this time period, which might be quite acceptable in metabolic alkalosis, in contrast to the respiratory alkalosis. In the case of respiratory alkalosis, the use of oxygen therapy methods would effectively normalize the level of biochemical abnormalities.

The participation of the respiratory component in ABS disturbances is reflected by pO_2_ and pCO_2_ (Figure 1, Table 1). The change in the pCO_2_ index reflects a functional pathology of the respiratory system or is the result of compensatory reactions during metabolic shifts (9). pO_2_ can be defined as the oxygen pressure required to retain dissolved oxygen in arterial blood. The higher the pO_2_ is, the more oxygen there will be in blood and the higher the rate of movement of oxygen from capillary blood into the tissue (9). The average group pCO_2_ values in subjects with oxygen therapy were in a range below the standard values, but they did not have statistically significant differences from the standards (according to the results of a one-sample *t*-test). In CG, the average group pCO_2_ was lower than the standard ones (*t* = 3.4; *p* < 0.05). Higher pH values, together with the normal pCO_2_ values, can confirm the biochemical diagnosis of metabolic alkalosis. A tendency toward observing a decrease in pCO_2_ in the CG group may indicate a more serious condition (10), as these subjects did not receive additional oxygen therapy methods. The average pO_2_ levels in groups with oxygen therapy were in the range of the standard values, when using HBO from the first day of therapy and when using NOT from day 3 of therapy. In the CG, the mean group pO_2_ values were lower than the reference ranges throughout the entire treatment period and after a week of therapy did not reach the target norm (*t* = 2.9; *p* < 0.05). The average group values of SO_2_ in groups with oxygen therapy from the first day of AWS recovered to the standard values, which could not be seen in the CG group. In CG, the mean group values of SO_2_ were lower than the reference ranges throughout the entire treatment period (*t* = 4.1; *p* < 0.05). To improve SO_2_ values in the complex treatment of AWS, the HBO method was a priority, although the use of NOT also made it possible to restore SO_2_ from the first day of its use. The number of persons (%) with pCO_2_ under the normal values in CG was also greater (*p* < 0.05) than in groups with the oxygen therapy. It indicated that the oxygen therapy methods were more effective than only SDT in the correction of the severity of metabolic alkalosis in AWS. In groups with oxygen therapy, compared with CG, the number of subjects with low pO_2_ values decreased significantly (Supplementary Table 1). Therefore, the oxygen therapy methods in the complex AWS treatment, in comparison with SDT alone, reduce (from the first day of the treatment) more effectively the number of people with low saturation indices and increase the partial pressure of the oxygen. The advantages of methods of oxygen therapy over other SDT have not been established so far.

Total blood oxygen concentration (O_2_Ct) and oxygen content (O_2_Cap) starting from the first day of the AWS therapy were within the reference ranges in all the studied groups. The use of the HBO method in complex treatment more effectively influenced these indicators compared with the NOT method in combination with SDT and the use of SDT alone (Table 2 and Supplementary Table 2). As these values in all the groups were within reference ranges, it was not subject to more detailed analyses.

The D(A-a)O_2_ is a difference between the alveolar and arterial oxygen concentration. D(A-a)O_2_ is useful in identifying the source of hypoxemia. This measurement helps locate the problem as either intrapulmonary or extrapulmonary (21, 22). According to Table 2 and Figure 1, the use of oxygen therapy methods in the complex treatment of AWS makes it possible to reduce the D(A-a)O_2_ levels much more effectively from the first day of therapy, when compared with the use of SDT alone (*p* < 0.05). High values of the A-a gradient are due to impaired oxygen transfer/gas exchange. They are usually associated with diseases of alveolar membrane, diffuse connective tissue diseases, or inadequacy of the ventilation-perfusion ratio. A high alveolar-arterial oxygen gradient may indicate low oxygen tension in mixed venous blood, low cardiac output, high oxygen consumption, or low hemoglobin concentration (22). Considering the normal levels of hemoglobin (Table 1) as well as the absence of serious problems from the cardiovascular and respiratory system in the history of all the subjects, these factors should be excluded. One of the likely factors of increased D(A-a)O_2_ values might be a high oxygen demand in the oxidation processes (22). Therefore, the use of oxygen therapy methods in the complex treatment of AWS more effectively restores oxygen deficiency, normalizes the levels of D(A-a)O_2_, and consequently, regulates the metabolic processes after alcohol intoxication.

In our study, the use of SDT alone or in combination with oxygen therapy methods did not have a significant effect on the levels of hemoglobin, glucose, Ca^2+^, or Na^+^ throughout the entire period of care. Even though statistically significant differences in the hemoglobin levels were presented (Table 1), these values did not require further analysis because their values did not go beyond the reference ranges in almost all the subjects. It should be noted that, in all the studied groups, those subjects with Ca^2+^ levels below the reference ranges had no significant positive dynamics in its correction during AWS (Supplementary Table 1). Hypocalcemia can contribute to the development of neuromuscular excitability and convulsive syndrome, which, in some cases, is a kind of compensation mechanism for alkalosis since lactates are formed during tonic muscle tension. The possible reasons for a decrease in the level of ionized calcium can include hyperosmolar states with changes in pH, pancreatitis, traumatic brain injury, and a lack of vitamin D (4, 9, 11). In our study, we also noted that levels of ionized sodium (Na^+^) were within the normal range (Table 1) in the vast majority of subjects in all the groups, and the applied methods of treatment did not have a significant effect on it.

In the mixed ANOVA design, we showed significant interactions for time and groups in the pCO_2_ value with a significant time-by-group interaction and significant interactions for time and groups in the pH and O_2_Ct values with a strong tendency to significant time-by-group interaction. However, we noted statistically significant within-group changes in the *t*-test only for pH but not for pCO_2_ and O_2_Ct values (Table 1). The effect of time in the pH value had a higher effect size than for groups. A significant effect of only time for Na+ and a significant effect of only group for pO_2_, SO_2_, glucose, Ca^2+^, and HCO${}_{3}^{-}$ were found. The significant effect of time and group but without significant time-by-group interaction was found for hemoglobin, BEecf, BEb, SB, O_2_Cap, and D(a-A)O_2_. Although HCO^3−^ values had significant effect only for groups, a significant decrease in HCO^3−^ value only in the CG group and a lack of decrease in oxygen intervention groups (SG1, SG2) may point on the treatment effect. Therefore, in this case, the lack of effect (as in the case of other ABS markers) may not exclude in fact the effect of oxygen therapy (NOT and HBO). In contrast, it may even indicate a positive oxygen therapy effect.

## Limitations

Some limitations of this study may be due to the lack of the results of ABS markers in the healthy control group and before the treatment in the SG1, SG2, and CG groups, which could show normal levels of indicators in comparison to the treatment. Another limitation is that only male participants were recruited to the study. Other methodological limitations could also have an impact on our results. The data of our study were analyzed in two ways, namely, within-group and between-group differences. A comparison of intragroup indicators may indicate the possible influence of a method on certain indicators. Intergroup differences may point out the advantages of a particular method on the dynamics of the levels of a certain biological marker. As there were no correlations found between the CIWA-A scores and ABS markers, it might be due to the indirect effect of ethanol metabolites on ABS state and their indirect link with abstinence symptoms.

## Conclusion

We found that subjects with moderate AWS had metabolic alkalosis phenomena. The use of oxygen therapy methods in combination with SDT in comparison to SDT alone allows for faster and more efficient correction of the markers of the metabolic alkalosis, as evidenced by the dynamics of the pH, pCO_2_, and pO_2_ levels; more effectively restores the saturation levels, starting from the first day of the AWS therapy, with the relative priority of the HBO method; reduces the D(A-a)O_2_ levels much more effectively from the first day of therapy in comparison with the use of SDT alone and restores oxygen deficiency; and more effectively includes reserve capabilities of the body for the restoration of ABS mainly due to the bicarbonate (hydrocarbonate) buffer system. We can also conclude that the applied methods of treatment in all groups did not significantly affect the indicators of excess of bases, actual hydrocarbonate, and standard bicarbonate, which might be quite acceptable in metabolic alkalosis. Although the novel oxygen-related therapeutic procedures such as NOT and HBO in combination with standard drug therapy did not improve the alcohol withdrawal symptoms, it helped with the faster restoration of the ABS state. Further studies, with a control group before the oxygen therapy, should elaborate more extensively on the time and group effects of oxygenation on the AWS.

## Data Availability Statement

The original contributions presented in the study are included in the article/Supplementary Material, further inquiries can be directed to the corresponding author/s.

## Ethics Statement

The studies involving human participants were reviewed and approved by the Local Bioethical Committee of Belarusian State Medical University (N 8/124.15.08.2017). The patients/participants provided their written informed consent to participate in this study.

## Author Contributions

DK and AK: conceptualization. DK, AK, IK, and LK: data curation and formal analysis. DK, AK, and IK: investigation and methodology. AK: project administration and supervision. DK, AK, IK, NW, and LK: writing–original draft. NW: writing–review and editing. All authors contributed to the article and approved the submitted version.

## Funding

This study was supported in part by grants from Belarusian State Medical University (Belarus) and from Medical University of Bialystok (SUB/1/DN/21/001/1147, Poland).

## Conflict of Interest

The authors declare that the research was conducted in the absence of any commercial or financial relationships that could be construed as a potential conflict of interest.

## Publisher's Note

All claims expressed in this article are solely those of the authors and do not necessarily represent those of their affiliated organizations, or those of the publisher, the editors and the reviewers. Any product that may be evaluated in this article, or claim that may be made by its manufacturer, is not guaranteed or endorsed by the publisher.
