# Supplementary material for: The Effect of the Non-compressed Oxygen Therapy and Hyperbaric Oxygenation in Combination With Standardized Drug Therapy on the Blood Acid-Base State Biomarkers in Alcohol Withdrawal Syndrome, an Experimental Study
Source: Front Psychiatry. 2022 Apr 18;13:819154. doi: 10.3389/fpsyt.2022.819154 (PMC9058062; doi:10.3389/fpsyt.2022.819154)
Supplement: Supplementary file 1 [file Data_Sheet_1.docx]

**Title: The effect of the non-compressed oxygen therapy and hyperbaric oxygenation in combination with standardized drug therapy on the blood acid-base state biomarkers in alcohol withdrawal syndrome, an experimental study.**

Supplementary Material

# Supplementary Tables

**Table 1**. Distribution of subjects (%) with blood levels of the main indicators of acid-base state (ABS), deviating from the normal range (N), in the blood of persons suffering from alcohol withdrawal syndrome (AWS) that had standardized drug therapy (SDT), non-compressed oxygen therapy (NOT), and hyperbaric oxygenation (HBO).

| Index | Groups | | | р |
| --- | --- | --- | --- | --- |
|  | GS1 (1) | GS2 (2) | CG (3) |  |
| Number of persons (%) with indicators рСО_2_<N after 1 day of therapy | 61,5% | 62,7% | 79,7% | P_1,2-3_ <0,05 |
| Number of persons (%) with indicators рСО_2_<N after 3 days of therapy | 60,4% | 64,6% | 78,6% | P_1,2-3_ <0,05 |
| Number of persons (%) with indicators рСО_2_<N after 7 days of therapy | 56,2% | 55,3% | 74,3% | P_1,2-3_ <0,05 |
| Number of persons (%) with indicators рО_2_<N after 1 day of therapy | 75,0% | 57,1% | 74,2% | P_1,3-2_ <0,05 |
| Number of persons (%) with indicators рО_2_<N after 3 days of therapy | 56,0% | 45,8% | 82,8% | P_1,2-3_ <0,05 |
| Number of persons (%) with indicators рО_2_<N after 7 days of therapy | 54,2% | 42,6% | 83,3% | P_1,2-3_ <0,05 |
| Number of persons (%) with indicators SO_2_<N after 1 day of therapy | 14,3% | 14,0% | 33,3% | P_1,2-3_ <0,05 |
| Number of persons (%) with indicators SO_2_<N after 3 days of therapy | 16,3% | 17,5% | 50,0% | P_1,2-3_ <0,05 |
| Number of persons (%) with indicators SO_2_<N after 7 days of therapy | 12,5% | 11,4% | 50,0% | P_1,2-3_ <0,05 |
| Abbreviations: CG – patients who underwent SDT only (control group), GS1 – patients who underwent SDT in combination with NOT, SG2 - patients who underwent SDT in combination with HBO; pCO2 partial pressure of carbon dioxide in the blood, pO2 - partial pressure of oxygen in the blood, SO2 oxygen saturation; P1.2,-3<0.05 means statistically significant difference between the GS1 (1) and CG(3) groups, statistical difference between the SG2 (2) and CG(3) groups, and no differences between the SG1 (1) and SG2 (2) groups; P1.3-2 <0.05 means statistically significant difference between the SG1 (1) and SG2 (2) groups, statistical difference between the CG(3) and SG2 (2) groups, and no differences between the SG1 (1) and CG (3) groups; p<0.05 at the bottom of each group means statistically significant difference in the parameters during the seven-day treatment period. There were found intragroup differences only in the number of persons (%) with indicators рО_2_<N values -in SG1 and SG2 groups. | | | | |

**Table 2.** Distribution of subjects (%) with blood levels of the main indicators of acid-base state (ABS), deviating from the normal range (N), in the blood of persons suffering from alcohol withdrawal state (AWS) that had standardized drug therapy (SDT), non-compressed oxygen therapy (NOT), and hyperbaric oxygenation (HBO).

| Index | Groups | | | р |
| --- | --- | --- | --- | --- |
|  | GS1 (1) | GS2 (2) | CG (3) |  |
| Number of persons (%) with indicators HCO_3_- >N after 1 day of therapy | 86,5% | 86,0% | 65,4% | - |
| Number of persons (%) with indicators HCO_3_- >N after 3 days of therapy | 88,7% | 80,4% | 56,0% | P_1,2-3_<0,05 |
| Number of persons (%) with indicators HCO_3_- >N after 7 days of therapy | 88,0% | 83,0% | 60,0% | P_1,2-3_<0,05 |
| Number of persons (%) with indicators BEecf >N after 1 day of therapy | 92,5% | 94,0% | 86,2% | - |
| Number of persons (%) with indicators BEecf >N after 3 days of therapy | 90,4% | 93,6% | 79,2% | P_1,2-3_<0,05 |
| Number of persons (%) with indicators BEecf >N after 7 days of therapy | 90,4% | 86,7% | 79,2% | P_1,2-3_<0,05 |
| Number of persons (%) with indicators BEb >N after 1 day of therapy | 86,8% | 72,0% | 45,2% | P_1,2-3_<0,05 |
| Number of persons (%) with indicators BEb >N after 3 days of therapy | 81,1% | 72,3% | 29,6% | P_1,2-3_<0,05 |
| Number of persons (%) with indicators BEb >N after 7 days of therapy | 82,4% | 72,9% | 28,6% | P_1,2-3_<0,05 |
| Number of persons (%) with indicators SB >N after 1 day of therapy | 96,2% | 94,2% | 80,6% | P_1,2-3_<0,05 |
| Number of persons (%) with indicators SB >N after 3 days of therapy | 96,1% | 87,5% | 63,3% | P_1,2-3_<0,05 |
| Number of persons (%) with indicators SB >N after 7 days of therapy | 96,1% | 87,5% | 63,3% | P_1,2-3_<0,05 |
| Number of persons (%) with indicators D(A-a)O_2_ >N after 1 day of therapy | 72,5% | 68,2% | 100,0% | P_1,2-3_<0,05 |
| Number of persons (%) with indicators D(A-a)O_2_ >N after 3 days of therapy | 72,0% | 51,2% | 93,1% | P_1,2-3_<0,05 |
| Number of persons (%) with indicators D(A-a)O_2_ >N after 7 days of therapy | 64,6% | 59,5% | 93,1% | P_1,2-3_<0,05 |
| CG – patients who underwent SDT only (control group), SG1 – patients who underwent SDT in combination with NOT, SG2 - patients who underwent SDT in combination with HBO; BEb -blood base excess/actual base excess, BEecf -extracellular fluid base excess/base excess, HCO3 -bicarbonate level, SB -standard bicarbonate, D(A-a)O2 -alveolar-arterial gradient; P1.2,-3<0.05 means statistically significant difference between the SG1 (1) and CG(3) groups, statistical difference between SG2 (2) and CG(3) groups, and no differences between the SG1 (1) and SG2 (2) groups; p<0.05 at the bottom of each group means statistically significant difference in parameter during the seven-day treatment period. There were not found intragroup differences in the SG1, SG2 nor CG group. | | | | |
